# 

**Published:** 1789

**Authors:** 


### LONDON

### MEDICAL JOURNAL,

FOR THE YEAR 1789,

### PART THE THIRD.

### CATALOGUE OF BOOKS.

1. AN Account of the principal Lazarettos in Europe; with various Papers relative to the Plague: together with further Obſervations

* As the formation of teeth and hair involved in a fatty maſs may be ſaid to be peculiar to the ovaria, and as there are ſtrong reaſons for believing that this formation may take place without any intercourſe between the ſexes, it becomes difficult to account for this peculiarity in them, unleſs by ſuppoſing that they have a greater aptitude of running into ſuch a proceſs than the other parts of the body.

ſervations on ſome foreign Priſons and Hoſpitals; and additional Remarks on the preſent State of thoſe in Great Britain and Ireland. By *John Howard*, F. R. S. 4to. *Cadell*, London, 1789.

2. A Treatiſe on the Diſeaſes of Children, with general Directions for the Management of Infants from the Birth. By *Michael Underwood*, M. D. Licentiate in Midwifery of the Royal College of Phyſicians in London, and Phyſician to the Britiſh Lying-in Hoſpital. A new Edition, reviſed and enlarged; in two Volumes, 8vo. *Matthews*, London, 1789.

3. An Eſſay on Phlogiſton, and the Conſtitution of Acids. A new Edition. By *R. Kirwan*, Eſq. Member of the Academies of Stockholm, Upſal, Dijon, Dublin, Philadelphia, Mancheſter, &c. To which are added Notes, exhibiting and defending the antiphlogiſtic Theory, and annexed to the French Edition of this Work, by Meſſrs. *De Morveau*, *Lavoiſier*, *De la Place*, *Monge*, *Berthollet*, and *De Fourcroy*: tranſlated into Engliſh. With additional Remarks and Replies, by the Author. 8vo. *Johnſon*, London, 1789.

4. Aſiatic Reſearches; or, Tranſactions of the Society inſtituted in Bengal for inquiring into into the Hiftory and Antiquities, the Arts, Sciences, and Literature of Afia. Volume the Firft *. 4to. Calcutta, 1788.

5. Advice to Gouty Perfons. By Dr. *Kentifh.* 8vo. *Murray*, London, 1789.

6. Obfervations on the Brunonian Practice of Phyfic, including a Reply to an anonymous Publication reprobating the Ufe of Stimulants in Fevers. By *George Moffman*, M. D. 8vo. *Law*, London, 1788.

7. Memoirs of the Medical Society of London, inftituted in the Year 1773. Vol. II. 8vo. *Dilly*, London, 1789.

8. The Inftruments of Medicine; or, The Philofophical Digeft and Practice of Phyfic. By *George Hoggart Toulmin*, M. D. 8vo. *Johnfon*, London, 1789.

9. A Lecture on the Atmofphere of London, as read before a public Society, June 14, 1788; with four Plates illuftrative of the Phenomena, and a Preface. By *Benjamin Taylor*. 4to. *Dilly*, London, 1789.

* We meet with two papers on chemical fubjects in this volume, viz. 1. Of the method of diftilling as practifed by the natives of Chatra in Ramgur; by Archibald Keir, Efq. 2. The procefs of making *Attar*, or Effential Oil of Rofes; by Lieutenant-colonel Polier.

 10. Hif-

10. Hiſtory of ſome of the Effects of Hard Drinking. By *J. C. Lettſom*, M. D. F. R. S. and F. S. A. 4to. *Dilly*, London, 1789.

11. The Works of the late *John Gregory*, M. D. 4 Vols. 8vo. *Cadell*, London, 1788.

12. Diſſertatio Inauguralis Medica de Tartari Emetici præparatione et viribus medicis; Auctore *Chriſtian. Frider. Witting*, Eimbecca-Hannoverano. 8vo. Gottingæ, 1788.

13. Amphibiorum virtutis medicatæ Defenſio inchoata quam Præſide *Johanne Hermann*, Doct. et Prof. Med. Publ. Cap. Thom. Can. ſolemniter defendet die 29 Martii, A. 1787, *Joannes Godofredus Schneiter*, Argentinenſis. 4to. Argentorati, 1787.

14. Amphibiorum virtutis medicatæ Defenſio continuata, Scinci maxime Hiſtoriam expendens; quam Præſide *Johanne Hermann*, Med. D. &c. ſolemniter defendet die 4 Aprilis, 1789, *Jacobus Fridericus Schweighæuſer*, Argentinenſis. 4to. Argentorati, 1789.

15. *Dominici Vandelli*, Academiæ Regalis Scientiarum Oliſoponenſis Socii, &c. Viridarium Griſley Luſitanicum, Linnæanis nominibus illuſtratum, juſſu Academiæ in lucem editum. 8vo. Oliſipone, 1789.

16. *Dominici Cyrilli* in Neap. Lyceo Med. Theor.

Theor. Prof. &c. &c. Plantarum rariorum Regni Neapolitani Fasciculus primus cum Tabulis æneis. 4to. Neapoli, 1788.

17. Abhandlung ueber die Venerische Krankheit; von *Christoph Girtanner*, der Arzneiwissenschaft und Wundarzneikunst Doctor, der Koenigl. Societaet der Wissenschaften zu Goettingen Correspondenten. 8vo. Gottingen, 1789. Vols. II. and III.

These two volumes, to which the work has been unavoidably extended, complete the author's plan *. They contain a very curious and (so far as we are able to judge) accurate review, in chronological order, of more than eighteen hundred different publications on the venereal disease. At the end of the third volume Dr. Girtanner has given copious extracts, relative to this subject, from the earliest (chiefly Spanish) writers on the History of America.

18. Das Allgemeine Krankenhaus in Mainz, &c. *i. e.* The General Hospital at Mentz described by *Charles Strack*, M. D. Professor of Physic in the University of Mentz, &c. 8vo. Franckfort on the Main, 1788.

* See Vol. IX. page 427.

